# Patients know best: DESTINIES Patient Panel appraisal of a novel, risk-stratified approach to COVID-19 disease surveillance in the adult immunosuppressed

**DOI:** 10.1093/oxfimm/iqag015

**Published:** 2026-09-18

**Authors:** Meredith Leston, Ingrid de Groot, José M Ordóñez-Mena, F D Richard Hobbs, Mark Joy, Lennard Y W Lee, Benjamin W Teh, Iain McInnes, Simon de Lusignan

**Affiliations:** Nuffield Department of Primary Care Health Sciences, University of Oxford, Oxford, OX2 6GG, United Kingdom; Dutch Myositis Working Group, Spierziekten Nederland, Lt.Gen van Heutszlaan 6, Baarn 3743JN, Netherlands; Nuffield Department of Primary Care Health Sciences, University of Oxford, Oxford, OX2 6GG, United Kingdom; Nuffield Department of Primary Care Health Sciences, University of Oxford, Oxford, OX2 6GG, United Kingdom; Nuffield Department of Primary Care Health Sciences, University of Oxford, Oxford, OX2 6GG, United Kingdom; Department of Oncology, University of Oxford, Old Road Campus Research Building, Roosevelt Drive, OX3 7DQ, United Kingdom; Department of Infectious Diseases, Peter MacCallum Cancer Centre, 305 Grattan Street, Melbourne, VIC 3000, Australia; MVLS College Office, Wolfson Medical School Building, University of Glasgow, University Avenue, Glasgow, G12 8QQ, United Kingdom; Nuffield Department of Primary Care Health Sciences, University of Oxford, Oxford, OX2 6GG, United Kingdom

**Keywords:** COVID-19, immunosuppression, disease surveillance, infectious disease, patient engagement

## Abstract

The immunosuppressed are poorly represented in disease surveillance and pharmacovigilance dataflows, proving especially consequential during the COVID-19 pandemic. This mixed methods study recruited a panel of immunosuppressed patients (DESTINIES Patient Panel) to quality assure a new digital phenotype for COVID-19 risk in the adult immunosuppressed (DESTINIES phenotype) prior to its implementation in real-world surveillance. Applying their lived experiences, the DESTINIES Patient Panel met to critique the strategy and structure of the provisional phenotype before voting on its adoption in UK disease surveillance. They did so anonymously. This convening was hosted and recorded on Zoom; Google Forms collected panellist feedback and their final ratifying vote. Feedback was inductively content analysed. 33 patients that adhered to the DESTINIES phenotype definition of immunosuppression submitted their feedback and final vote. Despite obtaining expressions of interest, no person with drug-managed HIV participated; this was a significant limitation given their unique positioning in the phenotype. However, 88% of panellists approved the DESTINIES phenotype for adoption in the UK, 91% agreed it represented a step forward for COVID-19 disease surveillance, and 91% agreed to see it trialled in other surveillance pathways (RSV, pneumonia etc.). Panellist feedback was catalogued by themes of ‘Abandonment’, ‘Accessibility’, ‘Accuracy’, and ‘Omission’. The patient voice is often absent in the dataflows purporting to be in their interest. This is an oversight; certain limitations captured in this exercise were unlikely to have surfaced without patient engagement. The DESTINIES phenotype is made more robust by the collective criticism of expert clinicians and patients alike.

## Introduction

Disease surveillance and pharmacovigilance are integral to the success of health systems [1]. Dataflows can detect emerging or novel threats [2], the spread of disease in a community [1], and audit the ongoing benefit-risk profile of pharmaceutical products [3]. They are assets for pandemic preparedness [4], especially in an era of artificial intelligence-augmentation and advanced clinical informatics [5, 6]. However, at present, these pathways are usually produced and released without patient consultation or endorsement [7]. Given the highly sensitive nature of surveillance and increasingly fraught public health landscape, this is both a missed opportunity and potential contributor to misinformation and institutional mistrust [8–10].

Moreover, disease surveillance and pharmacovigilance pathways do not capture the experiences of clinical risk groups at sufficient resolution to inform targeted care [11]. This is especially notable for immunosuppression, where patient data is conventionally recorded, analysed, and reported in aggregate [12], or is exclusively differentiated by dominant subgroups [13]. In either respect, these dataflows risk erasing critical sub-trends and are ineffectual for needs-based disease prevention or response [12], Homogenised approaches have become the norm for protecting vulnerable patients as a result–a strategy that is detrimental to health outcomes [13].

The COVID-19 pandemic has proven instrumental for challenging this approach to immunosuppressed disease surveillance and public health delivery: without targeted data on the varied COVID-19 infection and vaccination experiences of the immunosuppressed, universalised disease prevention and treatment plans put lives and livelihoods at risk [12]. The convention of excluding these patients from clinical development trials [14], including those informing the emergency authorisation of COVID-19 vaccination [15], exacerbated poor pharmacovigilance amongst this vulnerable group and contributed to vaccine hesitancy and refusal despite the risks associated [16].

The eDElphi STudy to fully defiINe and COVID-risk stratify ImmunosupprESsion (DESTINIES Study) [17] was conducted to improve the representation of the immunosuppressed within disease surveillance. It brought together international experts within vaccinology, immunology, and infectious disease to develop and ratify a COVID-19 risk stratified digital phenotype for adult immunosuppression. The DESTINIES phenotype that surfaced through this consensus building exercise (Fig. 1, within) is a tool capable of extracting and comparing immunosuppressed COVID-19 data by historical risk for severe infection outcomes. This produces higher resolution insights and vigilance than prevailing approaches. It achieved 94% expert approval (*n* = 34) for immediate operationalisation in real-world COVID-19 dataflows.

**Figure 1:**
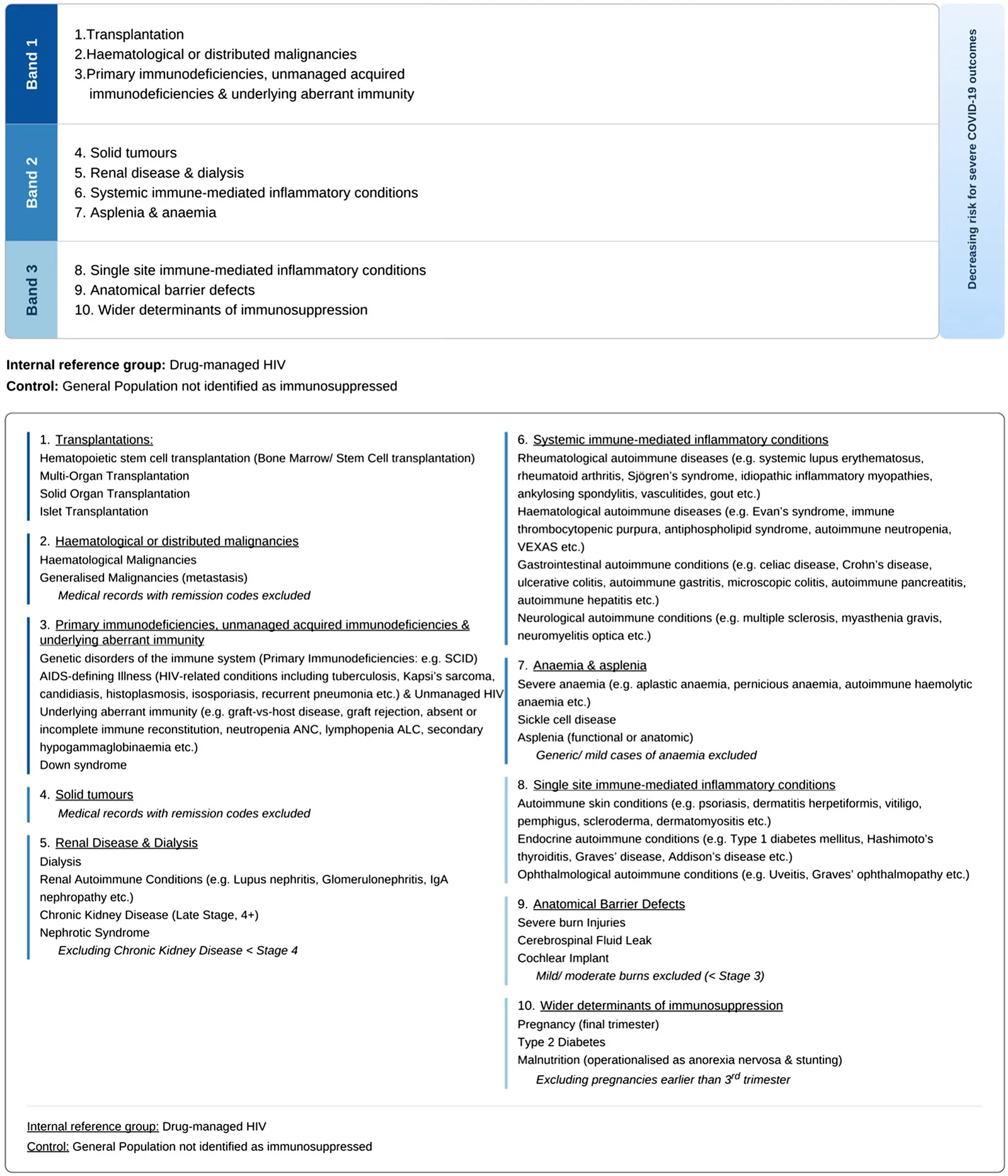
The DESTINIES phenotype, illustrated and operationalised.

However, this phenotype has yet to be evaluated or endorsed by the immunosuppressed themselves. As such, the present investigation aimed to recruit a panel of all patient populations incorporated in the DESTINIES phenotype (the DESTINIES Patient Panel) to allow for direct patient critique, refinement and approval of this new surveillance tool prior to implementation in UK national dataflows. Doing so offered an additional check on its real-world applicability and acceptability.

## Method

### Design

The DESTINIES steering committee, described elsewhere [17] and inclusive of an immunosuppressed patient champion, oversaw the development of this study and the clinical expert eDelphi that informed it. Said patient champion was connected to this work by the European Alliance of Associations for Rheumatology (EULAR); they were instrumental in study governance, assuring the patient-centricity of recruitment strategy and all study materials, as well as data collection, interpretation, and dissemination.

A mixed methods design was utilised in this work, applying inductive content analysis to understand patient perspectives on the prospective strengths and weaknesses of the DESTINIES phenotype for immunosuppressed COVID-19 disease surveillance (Fig. 1). A consensus vote aimed to ratify or reject its implementation in national dataflows.

### Recruitment

Recruitment for the DESTINIES Patient Panel was global and purposive, fielding expressions of interest until representatives from every immunosuppressed subgroup identified in the DESTINIES phenotype had formally registered. The primary investigator and the patient champion led this process, advertising the opportunity within the National Institute of Health Research (NIHR)’s *People in Research* portal [18] and amplifying the call via the European Alliance of Associations for Rheumatology (EULAR) People with Arthritis/Rheumatism in Europe (PARE) community [19] as well as leading immunosuppressed advocacy and affinity organisations.

Registered patient panellists were informed that a 1-hour webinar—hosted on the Zoom (Zoom, inc) video conferencing platform—would be arranged to introduce them to key concepts in disease surveillance, including digital phenotyping, and give them the opportunity to critique plans for immunosuppressed-specific changes. A Google Form (Alphabet) was distributed to identify the most popular date for the webinar and to obtain initial consent for recording the session and disseminating results via academic publication. Patients were informed that their participation would be anonymised and that, subsequent to the webinar, they would be invited to vote on a new system of immunosuppressed disease surveillance. This information was reiterated in the consent forms that were distributed to those registered for the most popular webinar date. A copy of this consent form is available in Supplementary Materials. To support anonymisation, those confirmed for in-person webinar attendance were allocated unique panellist identification numbers (IDs) and were provided with detailed instructions on disabling their cameras and changing their Zoom participant names to match their unique ID.

### Webinar content and feedback

Titled, ‘*Improving how we monitor COVID-19 spread and outcomes in the immunosuppressed’,* the webinar presented the origins of the DESTINIES phenotype, including outputs from the literature reviews and the eDelphi study that informed its structure and content, as well as the outcomes of its pilot implementation in real-world data. The primary investigator and patient champion worked together to ensure the accessibility of all presentation materials and messaging. This process pulled upon Alley’s ‘The Craft of Scientific Writing’ (1996) [20], giving due consideration to presentation audience, purpose, and mode to improve communication and understanding. The careful use of personal disclosure and pertinent analogies aimed to build rapport between the presenting primary investigator and the patient panel–eliminating power dynamics while keeping content engaging. For transparency, presentation slides are available in Supplementary Materials. This content was shared for half an hour; remaining time fielded patient panellists’ criticisms and questions. The patient champion was available to ensure all primary investigator answers were sufficiently clarifying for patients.

To maximise engagement and feedback, this webinar was recorded and circulated to all patients who had expressed an interest in participating but were unable to attend live. A Google forms-based questionnaire accompanied this file and collected panellists’ final feedback on the DESTINIES phenotype and voting on its readiness for implementation in real-world dataflows. With patient champion oversight and piloting, the question set and final vote that ratified the DESTINIES phenotype amongst expert clinicians [17] were repurposed for this patient feedback to ensure coherence between the two exercises. The full question set is available in Supplementary Materials, including free-text items (‘*Please write down any questions you have about the research we presented, the DESTINIES phenotype or our future plans*’ and ‘*Please write down any feedback you have on the research we presented and our DESTINIES phenotype’*) and those appraised by Likert scale (Strongly Disagree, Somewhat Disagree, Unsure, Somewhat Agree, Strongly Agree). As per the DESTINIES Study that preceded this exercise and Diamond *et al*. (2014) [21] guidance, patient panel consensus was pre-determined at ≥ 75% alignment on Likert poles (Strongly Disagree and Somewhat Disagree or Strongly Agree and Somewhat Agree). Likert-based items included ‘*I think this phenotype should be used in COVID-19 disease surveillance moving forwards’*, ‘*I think this phenotype represents a step forward for immunosuppressed COVID-19 disease surveillance’,* and ‘*I would like to see this phenotype trialled in the surveillance of other diseases* (e.g. *seasonal flu, pneumonia etc.*)’.

Panellists had ongoing access to the webinar recording and were given two weeks to submit their responses to the Google Form-based questionnaire supplied. Unifying themes of webinar commentary and free-text questionnaire item responses were identified via inductive content analysis. Exemplifying quotations were reported.

### Ethical approvals

Prior to patient panel recruitment, the primary investigator sought ethical approval for this exercise from the University of Oxford Research Governance Ethics and Assurance Team. However, as per the DESTINIES study [17], this study was categorised as ‘pre-research, priority setting or survey’ and thereby inappropriate for formal review–consistent with the Department of Health’s UK Policy Framework for Health and Social Care Research, [22] the Health Care Quality Improvement Partnership’s *Guide for Clinical Audit, Research and Service Review* [23] and Health Research Authority’s review algorithm and attendant leaflet (*Defining Research*) [24].

## Results

### Descriptive analysis

This investigation was conducted between April and July of 2025. 25 immunosuppressed patients attended the convening remotely. After distributing the presentation file, meeting recording and Google Form for feedback and voting to all those who had expressed interest in participating, a total of 33 immunosuppressed patients submitted their responses. The demographic composition of this cohort is tabulated in Table 1; patients were able to disclose multiple diagnoses where relevant.

**Table 1:** Characteristics of the DESTINIES patient voting cohort

	Variable	*n* = 33
**Sex**	Male	6
	Female	26
	Other	1
**Country**	India	1
	United Kingdom	27
	USA	2
	Czech Republic	1
	Netherlands	1
	Switzerland	1
**Immunosuppression**	Autoimmune disease	16
	Cancer	11
	Nutritional deficiency/malnutrition	2
	Transplantation	5
	Primary immunodeficiency	4
	Renal disease	1
	Rheumatological disease	7
	Other	2

Participants primarily came from the UK. Male patients were under-represented in this patient panel whereas autoimmunity and cancer were over-represented. Although many individuals with drug-managed HIV expressed an interest in participating, none contributed data to this exercise; all other patient groups denoted as immunosuppressed by the DESTINIES phenotype participated, however.

### Qualitative analysis

For transparency, all comments and questions raised during the live webinar are presented verbatim in Panel 1.

Panel 1 Unedited comments and questions raised by patient panellists during ‘*Improving how we monitor COVID-19 spread and outcomes in the immunosuppressed’* webinar

- ‘*There is no wastewater surveillance in UK’*
- *‘Immunosuppressed people are being disproportionally higher negatively impacted’*
- *‘What is definition of immunosuppressed?’*
- *‘Will this be useful in future pandemics or is it [COVID]-19 specific?’*
- *‘What is your estimation of number of [Immunosuppressed] people in UK? There seems to be a mixture of Figs reported…’*
- *‘Where do you get your info/data from; hospitals, GPs?’*
- *‘Are you using AI/ML to develop this digital phenotype?’*
- *‘Are you linking up with [STRAVINSKY] study?’*
- *‘Does the broad definition of [Immunosuppression] only include people on immunosuppressive medication?’*
- *‘With your definition of [Immunosuppression], have you created a scale,* i.e. *which groups are more [Immunosuppressed] than others- and does this equate to risk levels?’*
- *‘Clin[ical diagnosis] will be more or less equivalent internationally even if the definition of [Immunosuppression] is not.’*
- *‘Can I be included in/informed in future work on DESTINIES?’*

Inductive content analysis was applied to all free-text data collected via Google Forms. The following thematic categories were most representative of this qualitative feedback: ‘Abandonment’, ‘Accessibility’, ‘Accuracy’, and ‘Omission’. These are elaborated upon in turn, with exemplifying pull quotes provided (also verbatim).

#### Theme 1: abandonment

Praise for the DESTINIES phenotype was framed by the extent to which it addressed patients’ feelings of abandonment and raised awareness for their ongoing vulnerability to COVID-19; current practices were perceived as neglect. Patients shared their frustration with disease surveillance and care pathways that failed to accommodate their unique risks and, by contrast, their hope in the DESTINIES phenotype for delivering the needs-based advice on treatment options and reintegration with society long since advocated for.

‘It was an interesting presentation. I am glad to see your focus on monitoring [COVID]-19 in immunocompromised people. In many countries they still remain a neglected population.’

‘The level of detail in the DESTINIES data was very impressive. I hope that relevant organisations and authorities can use the information to improve the quality of care for immunocompromised people in the future, and also use your data to re-examine what were—in my opinion—inadequate and inconsistent care and protection measures for the immunosuppressed during the [COVID]-19 pandemic.’

‘The future plans are so exciting; I look forward to implementation of this project and works’

‘I think your work is critical as immunosuppressed patients need to be able to manage risk’

‘Extremely enlightening, the more information immunocompromised patients have, the more they can make choices around [COVID].’

Several patients were shocked to learn that the disaggregated and risk-stratified approach offered by the DESTINIES phenotype was not already standard practice.

‘I think it’s a great idea. On the one hand I’m very surprised that this type of data isn’t available already. Having said that my household feels forgotten. We are still wearing FFP3 masks at medical appointments to protect ourselves. My partner is still working from home 5 years on to protect me. The world needs to know that many of us are still living with the fear of Covid and possible consequences of catching it. I’ve just had my 11th [COVID] jab.’

There was clear appetite amongst the panellists to see this segmented evidence base shared with policy and pharmaceutical stakeholders and actioned to improve COVID-19 interventions and therapies. Partnerships with other research groups were also encouraged to scale the impact of this initiative.

‘I think it would be very useful to have the Destinies phenotype to try and adapt vaccines and treatment of the immune suppressed we are a forgotten group; people need to understand that our own bodies cause us damage and if there is a system that aids us in any way is of benefit’

‘I think it would be good to link into the STRAVINSKY Study; if your phenotype can be used at local level it could potentially be very useful to individuals trying to navigate their way through daily life in the current situation where [COVID] is over’ makes life difficult’

Patient panellist criticisms of the DESTINIES phenotype anchored around the themes that follow.

#### Theme 2: accessibility

Patients expressed concern that the DESTINIES phenotype would direct and prioritise immunosuppressed access to medications in the first instance–only creating more barriers for absent, atypical, or lower-risk patient types.

‘I would be concerned about the NHS using your data as an excuse to ration access to relevant treatment/prevention especially with regard to Covid but potentially future population risk.’

Researchers had to reassure these panellists that this work was solely intended for data generation–segmenting disease surveillance pathways to surface higher resolution insights on COVID-19 spread and outcomes in the immunosuppressed–and would not be used to reallocate resources.

Patient panellist feedback also indicated that, despite best efforts to the contrary, the webinar itself was sometimes too specialised for panellists to understand. Blurred slides and the omission of a patient-oriented glossary were also criticised.

‘The diagrams were too small to see well.’

‘Presentation was good but unfortunately the screen show was very small and not easy to read or follow.’

‘A simplified poster-presentation in lay language could be circulated to patient groups (2 were mentioned) for informing their members. A simplified tickbox survey could be used to elicit broad brush feedback on utility of the phenotype should the research continue’

‘It would have been useful to have a lay summary of the research to date, the next steps and the project endpoints. Also, a glossary of terms in the presentation circulated.’

‘To be honest, I think the presentation may have been difficult for some patients without a scientific background to follow, but I might be wrong here.’

#### Theme 3: accuracy

Patient panellists understood the prospective value of the DESTINIES phenotype for improving the resolution of immunosuppressed COVID-19 surveillance and, by extension, the accuracy of vaccine benefit-risk assessments that had previously been lacking.

‘Hopefully your research will help the NHS/NICE etc. to get more drugs like Evusheld given to patients who need it quicker than the current system [. Also] Paxlovid or drugs similar[:] we need a better system to be given these drugs especially at weekends and bank holidays and many GPs don’t understand that patients who are immune compromised/immune suppressed need to get the medication quickly’

[As previously] ‘The level of detail in the DESTINIES data was very impressive. I hope that relevant organisations and authorities can use the information to improve the quality of care for immunocompromised people in the future, and also use your data to re-examine what were—in my opinion—inadequate and inconsistent care and protection measures for the immunosuppressed during the Covid-19 pandemic.’

However, patient panellists also pointed out how the simplistic and exclusively diagnostic and procedural approach of the DESTINIES phenotype for COVID-19 risk stratification could create new inaccuracies or incorrect inferences. Single risk estimates for extremely heterogeneous subgroups were especially controversial for patients.

‘When presenting the phenotypes, it would be useful to explain that just because they are population phenotypes, it doesn’t necessarily follow that people with the worst phenotypes will personally do really badly if they get C19 (could be quite disheartening!)’

‘More detailed information on inherited Primary Immune Deficiency [is needed] in relation to your work.’

Certain patients also questioned founding assumptions of the DESTINIES phenotype, notably whether the immunosuppressed are conventionally excluded from pharmaceutical research and development pathways.

‘Also, I don’t really fully agree with the statement that Immunosuppressed patients are often excluded from clinical development trials, I think it would be good to give examples of this and how and what such trials consider as “immunosuppressed” and define in their exclusion criteria. My personal experience is that when I had the COVID-19 vaccinations and because I was on rituximab already, the assumption was that I would not produce antibodies. However, I produced a good amount to the contrary. I think it is really difficult to apply a phenotype when there is not yet a consensus on the definition of what is immunosuppressed.’

#### Theme 4: omission

Despite considering all medical diagnoses and procedures cited in international clinical criteria for immunosuppression, patients highlighted key omissions in the DESTINIES phenotype.

‘What measures have been taken to ensure this phenotype leaves no one behind?’

‘It would be interesting to consider if mast cell activation syndrome (MCAS) would also fall under the umbrella here or if it would be separate. As someone living with MCAS, I have to take a considerable amount of medication to calm my mast cells—but they are pretty quick to react to both vaccinations and infections (including the one time I’ve had COVID-19) on top of my [rheumatological] & [dermatological] conditions.’

Patients also highlighted the limited scope of the DESTINIES phenotype in its current form. Its COVID-19 specificity limits its generalisability to risk-stratifying broader conditions, and its static nature does little to appropriately categorise those with multi-morbidities or contextual factors, including medications, that might amplify or modify risk. The international applicability of this tool was also questioned.

‘Is there any scope for your work to be extended beyond the UK? Can the prototype of your work for Covid 19 be extended to other pandemics in future?’

‘How will this assist doctors keep IS patients safe during future outbreaks? Will the data be able to be extrapolated beyond COVID and/or the model used for other outbreaks?’

‘I appreciate that the [DESTINIES] Phenotype offers a more granular approach to stratifying those who are immunocompromised. However, practically, a more holistic approach is needed. The immunocompromised would like vaccines to be offered to their immediate families. There are specific needs for children. There is an urgent need for non-vaccine pre-exposure prophylaxis for those who do not respond to vaccines, and a need for development of such drugs to be fast-tracked in the same way that the vaccines were. Your research was interesting but I struggle to see how it is going to change existing approaches to the immunocompromised (which are inadequate).’

‘Can the results of the study be used in other countries (EU). What are the following steps after the [DESTINIES] study?’

### Quantitative analysis

Results from Likert scale-based questionnaire items are tabulated below. Poles are summed to assess panel consensus against the ≥ 75% criterion. Visualisations of these votes are provided in Fig. 2. Consensus agreement was achieved for all items, though not unanimously (Table 2).

**Figure 2:**
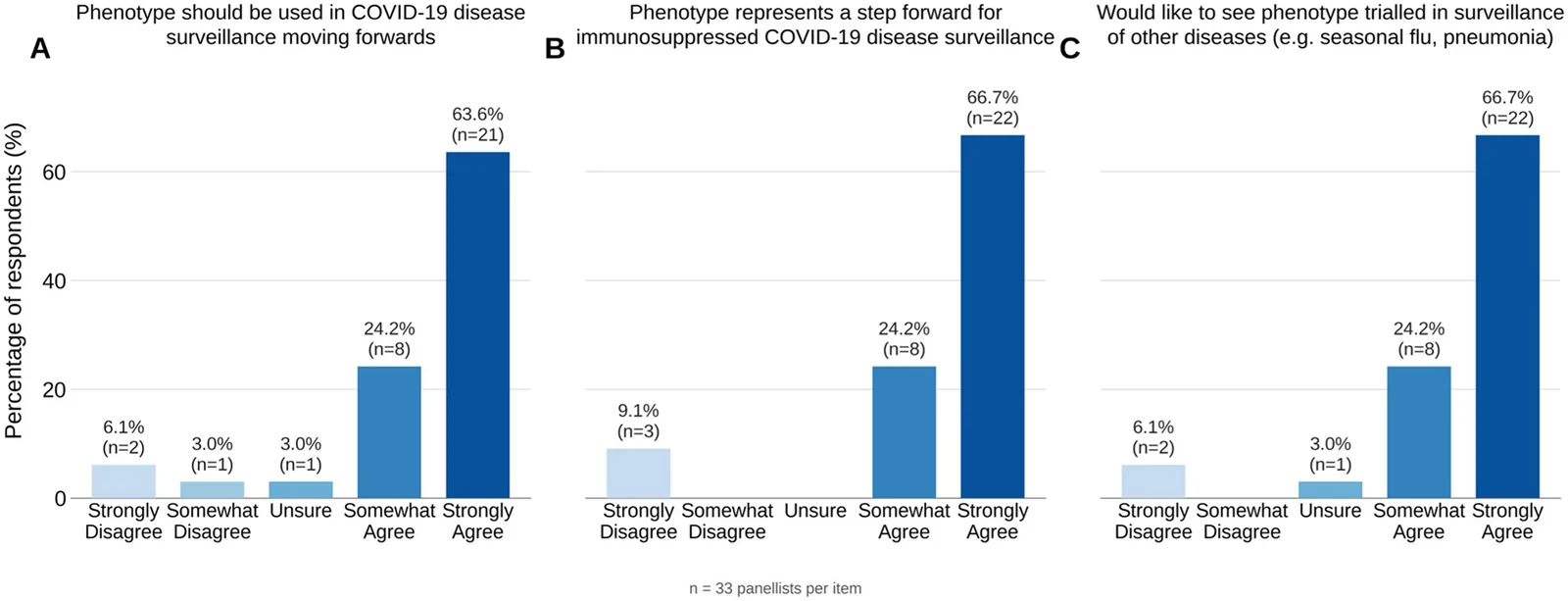
DESTINIES Patient Panel voting data.

**Table 2:** DESTINIES Patient Panel final ratification

	*A: ‘I think this phenotype should be used in COVID-19 disease surveillance moving forwards.’* (Panellist *n*)	%	B: ‘*I think this phenotype represents a step forward for immunosuppressed COVID-19 disease surveillance.’* (Panellist *n*)	%	***C: ‘I would like to see this phenotype trialled in the surveillance of other diseases (*** e.g.***seasonal flu, pneumonia etc.)’* (Panellist *n*)**	%
**Strongly Agree**	21	63.64	22	66.67	22	66.67
**Somewhat Agree**	8	24.24	8	24.24	8	24.24
***SUM Agree*** ^*^	***29***	***87.88***	***30.00***	***90.91***	***30.00***	***90.91***
**Unsure**	1	3.03	0	0.00	1	3.03
**Somewhat Disagree**	1	3.03	0	0.00	0	0.00
**Strongly Disagree**	2	6.06	3	9.09	2	6.06
***SUM Disagree*** ^**^	***3***	***9.09***	***3***	***9.09***	***2***	***6.06***

* Summation of Strongly Agree and Somewhat Agree.

** Summation of Strongly Disagree and Somewhat Disagree.

## Discussion

### Key findings

This mixed methods exercise convened a group of immunosuppressed patients to deliberate on the real-world value and risks associated with a novel phenotype for COVID-19 risk in the adult immunosuppressed (DESTINIES phenotype). The diverse diagnostic and international composition of this panel was to its credit. Said phenotype was successfully ratified for use in national disease surveillance; panellists also agreed to its superiority to prevailing surveillance dataflows and its prospective value for disease surveillance remits beyond COVID-19 (seasonal influenza, pneumonia etc). However, agreement was never unanimous, with certain panellists sharing significant concerns over the implementation of the phenotype in real-world data.

When content analysing webinar commentary, questions and questionnaire feedback inductively, themes of ‘Abandonment’, ‘Accessibility’, ‘Accuracy’, and ‘Omission’ were most salient. Patient panellists were receptive to the urgency of better segmented immunosuppressed disease surveillance and resultant care pathways but reflected on the potential risks associated with the crude and non-dynamic risk stratification that the DESTINIES phenotype affords in its current iteration. Key diagnostic and contextual omissions were also pointed out, including the absence of MCAS, comorbidity considerations, and validation data in other disease domains and lower income settings. However, overall patients commended this digital tool and expressed genuine excitement that the interests of the immunosuppressed would be better prioritised within disease surveillance moving forwards. Those who expressed misgivings were reassured when reminded of the surveillance specificity of this work; patients were emphatically opposed to seeing the DESTINIES phenotype dictate access to care.

### Comparison to wider literature

To the best of authors’ knowledge, this is the first patient engagement exercise that has informed the content, structure, and implementation of a digital phenotype for disease surveillance. Ratified by expert clinicians [17] and patients alike, the DESTINIES phenotype has undergone uniquely rigorous peer review and has benefitted substantially from the personal and professional experiences fielded.

Interestingly, there was a high level of alignment between the criticisms of these two consensus exercises: in both cases, attention was drawn to the static nature of the DESTINIES phenotype, its non-generalisability to diseases beyond COVID-19 and lower income settings, and its omission of key diagnoses or contextual determinants of risk. This, as well as the high levels of health literacy displayed by this patient panel, underscore the immense value of patient consultation for healthcare innovation–a resource that is, at present, under-utilised. Patient and clinician voting patterns also showed significant overlap.

That said, patient results deviated from clinical experts in important respects. For example, patients were slightly less favourable for DESTINIES phenotype implementation in real-world dataflows: 88% of patient panellists approved its operationalisation in COVID-19 pathways compared to 94% ratification (*n* = 34) amongst expert clinicians in the preceding eDelphi study [17]. Moreover, certain perspectives shared by patients likely would not have surfaced during expert clinician consultation–most notably the importance of timely and high-resolution disease surveillance for expediting the release of time-sensitive medications and supporting family members and caregivers of immunosuppressed individuals to align their own behaviour with community transmission levels.

Patient panellists’ appreciation for a more needs-based approach to disease surveillance is highly consistent with existing literature: patient advocates have long-since championed this issue and its potential for circumventing sparse clinical trial data and achieving more equitable care pathways and outcomes [25]. Likewise, patient panellists’ concerns that the purpose of the DESTINIES phenotype might easily be misunderstood or mischaracterised has strong precedent [26]. Disease surveillance is a highly specialised subject that is not intuitive for the public: for example, the use of R0, a metric of viral reproduction, to communicate the community spread of COVID-19 in the early phases of the pandemic produced more confusion than public coordination [27]. The term surveillance is also highly charged: disease surveillance pathways are not immune from politicisation [28]. In line with patient panellists’ criticisms, wider literature, and recent historical examples, this research and its intentions for enhancing disease surveillance must therefore be brought into the public domain with extreme care. Its intended pseudonymised outputs and public service motive must be communicated clearly and consistently.

Finally, this patient engagement exercise is far from the first to highlight the scientific value and rigour of the patient voice; similar exercises have seen patients successfully contribute to clinical guidelines, study protocols and community health interventions [29–32]. However, such efforts can quickly amount to tokenism if patient recommendations are not adopted or if patients are not updated on efforts to this effect. As such, researchers will now assess which criticisms reported in this exercise can be used to improve the DESTINIES phenotype prior to its implementation in national disease surveillance, and have committed to reconvening the patient panel to communicate these changes and present resultant research outputs as they become available.

### Limitations

This exercise would have greatly benefited from further patient champion consultation. While the contributions and oversight of our patient steering group member were invaluable to the accessibility and relevance of this investigation, additional expertise should have been sought out to maximise rigour and representativeness. Fielding input from dedicated patient research and interest groups from study inception would have likely avoided criticisms of poor accessibility, overly specialised language, and patient absences—notably, MCAS and other conditions now associated with post-acute sequelae of COVID-19 (PASC), or Long COVID [33, 34]. Doing so may also have improved the representativeness of panel composition; men were severely underrepresented in this work whereas certain patient groups, namely cancer and autoimmunity, were overrepresented.

Despite securing expressions of interest from all immunosuppressed subgroups represented in the DESTINIES phenotype, the absence of drug-managed HIV from the voting cohort was a key limitation of this exercise. The unique positioning of drug-managed HIV as an internal reference group makes this especially detrimental to the overall internal validity of this consensus exercise and the comprehensiveness of patient quality assurance. HIV remains a highly stigmatised condition [35], and it is possible that public participation in this panel–even if anonymised—was intimidating for those living with the condition. A separate consensus exercise, recruiting only drug-managed HIV patients, could be organised to compensate for this loss and ensure these patients endorse the DESTINIES phenotype before its integration into disease surveillance pathways as standard.

Furthermore, patients were only able to cross-reference preliminary real-world outcomes of the DESTINIES phenotype before making their ratifying votes, including the operationalisation of IMID levels and initial results of full operationalisation within a primary care trusted research environment (TRE). This affected their ability to fully appraise the prospective benefits and risks of routine implementation. Moreover, the summary forest plots patients were provided with (presenting phenotype levels’ raw and unadjusted COVID-19 outcomes compared to the immunocompetent) contained minor errors: confidence intervals were narrower than final outputs. The decision was made to not repeat this consensus exercise, however, as incidence rate ratio data was accurate and overall inferences remained the same. Instead, it is acknowledged that patients familiar with confidence intervals may have presumed greater accuracy in phenotype outputs than was the eventual reality–potentially biasing their evaluations.

Furthermore, our patient panellists’ engagement with both the NIHR *People in Research* portal [18] and a wide variety of advocacy, non-profit or affinity groups is suggestive of high levels of medical literacy - questioning overall representativeness of the general immunosuppressed public. This example of self-selection bias is typical within patient engagement exercises, however, and is difficult to control for in practice [36]. The poor representation of male patients seen in this work is also typical of public patient involvement and engagement (PPIE) and should have been counteracted with targeted recruitment [37]. Likewise, the UK-centricity of our panel should have been addressed prior to study commencement. These limitations undermine the external validity of our findings and should be addressed in future iterations or replications of this initiative.

Finally, this work did not catalogue the diversity of immunosuppressive medications patient panellists were receiving. Though justified at length elsewhere [17], the absence of medication-based forms of immunosuppression from both this exercise and the underlying DESTINIES Phenotype warrants criticism.

## Conclusion

This mixed methods work successfully recruited an international panel made up of diverse immunosuppressed patient representatives to quality assure and ratify a new means of monitoring the COVID-19 experiences of this clinical risk group. As a result, the DESTINIES phenotype is now endorsed by expert clinicians and patients alike for implementation in national disease surveillance pathways. Thanks to this joint oversight, it has since been successfully implemented in UK primary care data; results are forthcoming [38].

Moreover, the strong alignment between patient criticisms and those raised by clinical experts at a preceding consensus building exercise underscores the scientific rigour of patient engagement. Criticisms unique to the lived experiences of patients, meanwhile, demonstrate what is lost when the patient voice is excluded from clinical research and development pathways.

## Supplementary Material

iqag015_Supplementary_Data

## Data Availability

Study data and audio files can be made available upon request, subject to co-authorship review and approval from our patient panel.
