# Supplementary material for: Patients know best: DESTINIES Patient Panel appraisal of a novel, risk-stratified approach to COVID-19 disease surveillance in the adult immunosuppressed
Source: Oxf Open Immunol. 2026 Sep 18;7(1):iqag015. doi: 10.1093/oxfimm/iqag015 (PMC13651694; doi:10.1093/oxfimm/iqag015)
Supplement: Supplementary file 1 [file iqag015_supplementary_data.docx]

**DESTINIES Patient Panel Consent Form**

Meredith Leston

Nuffield Department of Primary Care Health Sciences

Radcliffe Observatory Quarter

Woodstock Road

Oxford

OX2 6GG

[Meredith.leston@phc.ox.ac.uk](mailto:Meredith.leston@phc.ox.ac.uk)

+447896980320

**CONSENT FORM**

**The DESTINIES Patient Panel Webinar: ‘Improving how we monitor COVID-19 spread and outcomes in the immunosuppressed’**

*Name of Researcher: Meredith Leston*

*If you agree, please put your initials in the box provided*

| 1. I understand that my participation in this webinar and subsequent vote is voluntary and that I am free to withdraw at any time without giving any reason. |  |
| --- | --- |
| 2. I agree to the webinar being audio recorded. |  |
| 3. I understand that findings from this work will be written up for publication in an academic journal. |  |
| 4. I agree to take part in this webinar and subsequent vote. |  |

| *_______________________* | *_________________* | *___________________________* |
| --- | --- | --- |
| *Name of Panellist* | *Date* | *Signature* |
| Meredith Leston | 18.04.25 |  |
| *Name of Person taking Consent* | *Date* | *Signature* |

**DESTINIES Patient Panel Feedback and Voting Form Question Set**

Online attendance, n = 25

Overall participation, n = 33

*Opportunity for DESTINIES Patient Panel to appraise DESTINIES Phenotype, ratifying its future use in national disease surveillance data flows.*

*Patient panellists were introduced to digital phenotyping, current approach to immunosuppressed disease surveillance, its limitations, and this work’s attempt to address them.*

*Patients were given the opportunity to ask any questions in real-time as well as via feedback and voting form (question set provided below).*

Name

Email

Country of residence

Gender

Class of medical condition

If you feel comfortable doing so, please specify the condition you have been diagnosed with. This information will be used to check we have obtained feedback from all those we hope to support through this work.

Please write down any questions you have about the research we presented, the DESTINIES Phenotype or our future plans.

Please write down any feedback you have on the research we presented and our DESTINIES Phenotype.

To what extent do you agree with the following statements about the DESTINIES phenotype? [I think this phenotype should be used in COVID-19 disease surveillance moving forwards. ]

To what extent do you agree with the following statements about the DESTINIES phenotype? [I think this phenotype represents a step forward for immunosuppressed COVID-19 disease surveillance.]

To what extent do you agree with the following statements about the DESTINIES phenotype? [I would like to see this phenotype trialled in the surveillance of other diseases (e.g. seasonal flu, pneumonia etc.)]

I understand that this work is part of an ongoing research project hosted by The University of Oxford.

I understand that outcomes of anonymised voting will be written up for publication in a scientific journal and doctoral thesis.

**DESTINIES Patient Panel Feedback During & After Webinar**

**Comments made and questions asked during webinar, ‘*Improving how we monitor COVID-19 spread and outcomes in the immunosuppressed’:***

- *‘There is no wastewater surveillance in UK’*
- *‘Immunosuppressed people are being disproportionally higher negatively impacted’*
- *‘What is definition of immunosuppressed?’*
- *‘Will this be useful in future pandemics or is it Covid-19 specific?’*
- *‘What is your estimation of number of IS people in UK? There seems to be a mixture of figures reported…’*
- *‘Where do you get your info/ data from; hospitals, GPs?’*
- *‘Are you using AI/MML to develop this digital phenotype?’*
- *‘Are you linking up with Stravinsky study?’*
- *‘Does the broad definition of IS only include people on immunosuppressive medication?’*
- *‘With your definition of IS, have you created a scale, i.e. which groups are more IS-ed than others- and does this equate to risk levels?’*
- *‘Clin.diagn. will be more or less equivalent internationally even if the definition of IS is not.’*
- *‘Can I be included in / informed in future work on DESTINIES?’*

**Questions asked via Feedback and Voting Form (unedited):**

*‘If it is adopted what is its future remit, can it continue to be developed and become more precise?’*

*‘How will the data be managed even within disease areas? There's been differing advice around immunisuppression even within disease areas and the impact of this in relation to covid 19.’*

*‘Is the research being published or presented in any conferernce papers, for example?’*

*‘Will the outcomes also be distilled and shared in formats which may be more comprehensible for those who are not familiar with interpreting statistical data or academic texts?’*

*‘It was mentioned that immunosuppressed people such as Solid Organ Transplant Recipients were excluded from the initial vaccine trials, yet this cohort was exactly the group who most needed protection, and for whom the vaccines were least effective. Many of us felt abandoned by this strategy, while seeing resources focussed on the wider population. Will your research encourage future vaccine developers and government health authorities to target resourses towards protecting the most vulnerable.’*

*‘What happens about multiple conditions - are any conditions additive in their risk eg lupus and hypogamma? Would a model that rabkws on highest risk miss an added risk? Also I’m assuming this model would obviously not be generalisable to other pandemic diseases depending on the profile and risk factors for that disease. Does this profiling include children with these conditions?’*

*‘I admit that I was disappointed with the webinar. I have been active in the Forgotten Lives group. Covid hit within a month of my transplant, and it took 3 years before my immune system recovered sufficiently to mount a response to the vaccines, so I was shielding for 3 years. Others are still shielding. We knew within a year that those with haematological malignancies were at highest risk for mortality, and that vaccines appeared ineffective for some. I had expected the research to address ways in which the immunocompromised could be recognised and helped more quickly in a future pandemic.’*

*‘What measures have been taken to ensure this phenotype leaves no one behind?’*

*‘Hopefully your research will help the NHS /nice etc to get more drugs like Evusheld given to patients who need it quicker than the current system, also paxlovid or drugs similar we need a better system to be given these drugs especially at weekends and bank holidays and many gps don’t understand that patients who are immune compromised/immune suppressed need to get the medication quickly, thank you again for your work’*

*‘Will your research be fed back to GP surgeries and local health authorities so that they can be better informed and provide a better service. Grateful for hospital treatment but GP surgeries could be so much better.’*

*‘Will patients be involved in your research going forwards?’*

*‘Will your work help to educate the media about Covid and its effect on the Immunocompromised. Long Covid is sometimes mentioned but immunocompromised have been forgotten.’*

*‘Will there be more meetings/work for us, PPI contributors?’*

*‘1. Is there any scope for your work to be extended beyond the UK?*

*2. Can the prototype of your work for Covid 19 be extended to other pandemics in future?’*

*‘How will this assist doctors keep IS patients safe during future outbreaks? Will the data be able to be extrapolated beyond COVID and/or the model used for other outbreaks?’*

*‘Will you link with the Stravinsky study which is looking at what levels of antibodies are required to have some protection and producing a test that can assess these levels easily similar to lateral flows.’*

*‘I would be concerned about the NHS using your data as an excuse to ration access to relevant treatment/prevention especially with regard to Covid but potentially future population risk.’*

*‘More information on inherited Primary Immune Deficiency in relation to your work’*

*‘Will they be further group talk on this topic’*

*‘Would your findings be used to collaborate with NHS to provide a clear pathway for obtaining antivirals?’*

*‘It would have been useful to have a lay summary of the research to date, the next steps and the project endpoints. Also a glossary of terms in the presentation circulated.’*

*‘A very ambitious and worthy project, will you be able to drill down to very small geographic areas?’*

*‘Can the results of the study be used in other countries(EU). What are the following steps after the Destinies study?’*

*‘I see you are working with Forgotten Lives, is this something you intend to carry on doing?’*

**Feedback provided via Feedback and Voting Form (unedited):**

*‘The presentation was excellent and gave a clear overview of the project.’*

*‘Very interesting and insightful. Happy to see more research taking place in this area.’*

*‘Brilliant, useful’*

*‘The level of detail in the DESTINIES data was very impressive. I hope that relevant organisations and authorities can use the information to improve the quality of care for immunocompromised people in the future, and also use your data to re-examine what were - in my opinion - inadequate and inconsistent care and protection measures for the immunosuppressed during the Covid-19 pandemic.’*

*‘I wondered how you were able to assess the risks faced by the immunospuppressed during Covid-19. The data shows that people in the high risk groups faced higher rates of hospitalisations and fatalities, but many of 'us' were also taking more extreme precautions than the general population to avoid becoming infected in the first place. Did the extra precautions result in lower infection rates for immunosuppressed people than might have been expected, and if so, could this have given an artificially lower impression of the actual infection risk?’*

*‘Anecdotally, I know of many transplant recipients who - like me - were still shielding, mask-wearing etc. 3 years+ into the pandemic, effectively ignoring the official guidelines telling us that we were at no greater risk than anyone eles. Like many others I know who are still exercising social precautions against infection, I have still never knowingly been infected with Covid-19, while others in the wider population report being infected multiple times. So, paradoxically those at least risk were apparently getting infected more easily, while those at high risk may not getting infected to the same extent. I wonder if this was something which is reflected in the data in the DESTINIES research?’*

*‘Very interesting and thank you for doing this.’*

*‘Presention was well thought through and put together, addressing things in logical order with flow. Interested to learn more and be involved if opportunities present themselves.’*

*‘I appreciate that the Destinies Phenotype offers a more granular approach to stratifying those who are immunocompromised. However, practically, a more holistic approach is needed. The immunocompromised would like vaccines to be offered to their immediate families. There are specific needs for children. There is an urgent need for non-vaccine pre-exposure prophylaxis for those who do not respond to vaccines, and a need for development of such drugs to be fast-tracked in the same way that the vaccines were. Your research was interesting but I struggle to see how it is going to change existing approaches to the immunocompromised (which are inadequate).’*

*‘To be honest, I think the presentation may have been difficult for some patients without a scientific background to follow, but I might be wrong here. Also, I don't really fully agree with the statement that Immunosuppressed patients are often excluded from clinical development trials, I think it would be good to give examples of this and how and what such trials consider as "immunosuppressed" and define in their exclusion criteria. My personal experience is that when I had the covid 19 vaccinations and because I was on rituximab already, the assumption was that I would not produce antibodies. However I produced a good amount to the contrary. I think it is really difficult to apply a phenotype when there is not yet a consensus on the definition of what is immunosuppressed.’*

*‘It would be interesting to consider if mast cell activation syndrome (MCAS) would also fall under the umbrella here or if it would be separate. As someone living with MCAS, I have to take a considerable amount of medication to calm my mast cells - but they are pretty quick to react to both vaccinations and infections (including the one time I've had COVID-19) on top of my rheum & derm conditions.’*

*‘Please keep going with your research for people who are immune compromised and the risks associated with COVID’*

*‘I think it's a great idea. On the one hand I'm very surprised that this type of data isn't available already. Having said that my household feels forgotten. We are still wearing FFP3 masks at medical appointments to protect ourselves. My partner is still working from home 5 years on to protect me. The world needs to know that many of us are still living with the fear of Covid and possible consequences of catching it. Ive just had my 11th Covid jab.’*

*‘Interesting presentation. It would have been to good to have a discussion in breakout rooms.’*

*‘It was an interesting presentation. I am glad to see your focus on monitoring Covid 19 in immunocompromised people. In many countries they still remain a neglected population.’*

*‘I think it's a valuable tool for the future’*

*‘I struggled to read slides 10, 11, 12 even after zooming in but I don't think it was too detrimental to my understanding of the general findings and recommendations.’*

*‘I think it could be increfibly useful, if it is generally accepted.’*

*‘The future plans are so exciting, I look forward to implementation of this project and works’*

*Some of the slides were blurred. I have emailed about the situation with regard to remission codes and use of this to exclude or include certain patients, therefore, accessing or not treatment pathways.’*

*‘I think your work is critical as immunosuppressed patients need to be able to manage risk’*

*‘It was an extremely interesting webinar.’*

*‘As above answer, more detailed information on inherited Primary Immune Deficiency in relation to your work.’*

*‘Good’*

*‘Extremely enlightening, the more information immunocompromised patients have, the more they can make choices around Covid.’*

*‘It would have been useful to have a lay summary of the research to date, the next steps and the project endpoints. Also a glossary of terms in the presentation circulated.’*

*‘When presenting the phenotypes, it would be useful to explain that just because they are population phenotypes, it doesn’t necessarily follow that people with the worst phenotypes will personally do really badly if they get C19 (could be quite disheartening!)’*

*‘A simplified poster-presentation in lay language could be circulated to patient groups (2 were mentioned) for informing their members. A simplified tickbox survey could be used to elicit broad brush feedback on utility of the phenotype and should the research continue’*

*‘Very good and beneficial to the immunosuppressed community.’*

*‘I think it woulld be good to link in to the Stravinsky Study; if your phenotype can be used at local level it could potantially be very useful to individuals trying to navigate their way through daily life in the current situation where 'Covid is over' makes life difficult’*

*‘Essential research - much needed’*

*‘Let participants of anonymized webinar change their avatars. I recognised someone.’*

*‘The diagrams were to small too see well.’*

*‘The study is very important. We should stop the Öne size fits them all" thinking. It may probably lead more public acceptance of the nessary measures.’*

*‘I think it would be very useful to have the Destinies phenotype to try and adapt vaccines and treatment of the immune suppressed we are a forgotten group, people need to understand that our own bodies cause us damage and if there is a system that aids us in any way is of benefit’*

*‘Presentation was good but unfortunately the screen show was very small and not easy to read or follow.’*

**Summary of Feedback**

- DESTINIES research was well-received by patient panellists – several expressed excitement that disease surveillance might now be better targeted to their interests. Patients agreed that this remit, and patient care more generally, was overly one size fits all – over-constraining the lives of some and underserving extremely high-risk patients.
- Patients were in complete agreement that more had to be done to make the world aware of the ongoing threat of COVID-19 to the immunosuppressed population. They were also deeply disturbed that such segmented surveillance was not already available as standard.
- Patients were eager to see this work rolled out to remits beyond COVID-19; the benefits of targeted surveillance and highly segmented evidence was clear to them for other disease domains and health use-cases. There were also suggestions to link DESTINIES work to other related study groups, including STRAVINSKY.
- Overall, the presentation was accessible enough for patients to understand the limitations of prevailing approaches to disease surveillance and the prospective value of the DESTINIES phenotype for overcoming them. Certain panellists would have benefitted from a glossary of key terms and enlarged visualisations in the slides that were distributed, however.
- Certain patients expressed concerns that this tool would be misinterpreted as an aid to clinical decision-making; given the immunosuppressed already face obstacles in accessing key medications, booster vaccine doses and enhanced medical attention, they emphasised the importance of communicating its surveillance-specificity to patients. They also worried that the caveats to DESTINIES exclusions (e.g. drug management of HIV and remission in certain cancers) would not be understood by patients; this would be especially detrimental to cancer survivors, many of whom still face heightened vulnerability to COVID-19 after receiving extremely immunosuppressive treatments when their disease was active. Finally, patients noted that Mast Cell Activation Syndrome patients were not mentioned explicitly – a segment of increasing importance due to associations with Long COVID – and questioned whether this phenotype captured heightened risk from multiple conditions and if it artificially suppressed immunosuppressed risk without controlling for increased cases in the non-shielded population.
- The patient panel communicated a keen interest in continuing to contribute to DESTINIES activities. They recommended that a one or two-page explainer be produced and distributed to all associated patient engagement groups to bolster panel membership as well as broader community awareness for the initiative – including amongst caregivers and clinicians.

**DESTINIES Patient Panel Final Ratification (n = 33)**

|  | **I think this phenotype should be used in COVID-19 disease surveillance moving forwards.** | **%** | **I think this phenotype represents a step forward for immunosuppressed COVID-19 disease surveillance.** | **%** | **I would like to see this phenotype trialled in the surveillance of other diseases (e.g. seasonal flu, pneumonia etc.)** | **%** |
| --- | --- | --- | --- | --- | --- | --- |
| Strongly agree | 21 | 63.64 | 22 | 66.67 | 22 | 66.67 |
| Somewhat agree | 8 | 24.24 | 8 | 24.24 | 8 | 24.24 |
| ***SUM agree*** | ***29*** | ***87.88*** | ***30.00*** | ***90.91*** | ***30.00*** | ***90.91*** |
| Unsure | 1 | 3.03 | 0 | 0.00 | 1 | 3.03 |
| Somewhat disagree | 1 | 3.03 | 0 | 0.00 | 0 | 0.00 |
| Strongly disagree | 2 | 6.06 | 3 | 9.09 | 2 | 6.06 |
| ***SUM disagree*** | ***3*** | ***9.09*** | ***3*** | ***9.09*** | ***2*** | ***6.06*** |
